# Gene expression of Hanwoo satellite cell differentiation in longissimus dorsi and semimembranosus

**DOI:** 10.1186/s12864-019-5530-7

**Published:** 2019-02-26

**Authors:** Sara de las Heras-Saldana, Ki Yong Chung, Seung Hwan Lee, Cedric Gondro

**Affiliations:** 1 School of Environmental and Rural Science, University of New England, Armidale, NSW Australia; 2 Hanwoo Research Institute, National Institute of Animal Science, RDA, Pyeongchang, South Korea; 3 Division of Animal and Dairy Science, Chungnam National University, Deajeon, South Korea; 4 Department of Animal Science, Michigan State University, 474 S Shaw Lane, East Lansing, MI 48824 USA

**Keywords:** Hanwoo, Satellite cell, Muscle differentiation, Myogenesis, RNA-seq

## Abstract

**Background:**

Korean Hanwoo cattle are known for their high meat quality, especially their high intramuscular fat compared to most other cattle breeds. Different muscles have very different meat quality traits and a study of the myogenic process in satellite cells can help us better understand the genes and pathways that regulate this process and how muscles differentiate.

**Results:**

Cell cultures of *Longissimus dorsi* muscle differentiated from myoblast into multinucleated myotubes faster than *semimembranosus*. Time-series RNA-seq identified a total of 13 differentially expressed genes between the two muscles during their development. These genes seem to be involved in determining muscle lineage development and appear to modulate the expression of myogenic regulatory factors (mainly *MYOD* and *MYF5*) during differentiation of satellite cells into multinucleate myotubes. Gene ontology enriched terms were consistent with the morphological changes observed in the histology. Most of the over-represented terms and genes expressed during myoblast differentiation were similar regardless of muscle type which indicates a highly conserved myogenic process albeit the rates of differentiation being different. There were more differences in the enriched GO terms during the end of proliferation compared to myoblast differentiation.

**Conclusions:**

The use of satellite cells from newborn Hanwoo calves appears to be a good model to study embryonic myogenesis in muscle. Our findings provide evidence that the differential expression of *HOXB2*, *HOXB4*, *HOXB9*, *HOXC8*, *FOXD1*, *IGFN1*, *ZIC2*, *ZIC4*, *HOXA11*, *HOXC11*, *PITX1*, *SIM2* and *TBX4* genes could be involved in the differentiation of *Longissimus dorsi* and *Semimembranosus* muscles. These genes seem to modulate the muscle fate of the satellite cells during myogenesis through a differential expression profile that also controls the expression of some myogenic regulatory factors (*MYOD* and *MYF5*). The number of differentially expressed genes across time was unsurprisingly large. In relation to the baseline day 0, there were 631, 155, 175, 519 and 586 DE genes in LD, while in SM we found 204, 0, 615, 761 and 1154 DE genes at days 1, 2, 4, 7 and 14 respectively.

**Electronic supplementary material:**

The online version of this article (10.1186/s12864-019-5530-7) contains supplementary material, which is available to authorized users.

## Background

The Korean Hanwoo cattle is known for its meat quality and high marbling ability (intramuscular fat) [1]. Meat quality (e.g. juiciness, tenderness, flavor) is mainly determined by the structure of the meat and its fatty acid composition, both of which vary widely across muscle groups [1–3].

Transcriptional analysis has been very useful to characterize gene expression differences in muscles from different breeds, with divergent phenotypes and across muscle groups [4]. It has also helped us understand the genetic mechanisms that underpin muscle development (myogenesis) [5] and how muscle developmental differences observed at the proteomic [6] and transcriptomic levels [7, 8] can affect production traits. Myogenesis is mainly controlled by the Myogenic Regulatory Factors (MRFs) that modulate myoblast proliferation, migration and fusion [9]. There are four MRFs (*MYF5*, *MYOD*, *MRF4/MYF6*, and *MYOG*), however there are also several other genes that contribute to the regulation of growth and differentiation [10] and there are still many unknowns surrounding the exact molecular mechanisms involved in muscle differentiation – particularly which genes change expression and when do these changes occur – that ultimately lead to the morphological and phenotypic differences observed across the different types of muscle.

Satellite cells are myogenic stem cells with the potential to self-renew and produce differentiated progeny; for this reason, these cells play an essential role in postnatal growth, muscle regeneration and hypertrophy. Myogenesis of satellite cells is a good model to study changes in gene expression over time and how they relate to muscle proliferation and differentiation [7, 8, 11, 12]. The combination of RNA sequencing with histological techniques allows for a deeper understanding of the mechanisms mediating differentiation of satellite cells into different muscle types revealing their gene expression profile and regulatory mechanisms at specific differentiation stages. A better understanding of these mechanisms is important for developmental biology and can assist in the development of therapeutic protocols in muscle.

In this study, muscle biopsies were performed on three Hanwoo calves to extract muscle satellite cells from *Longissimus dorsi* (LD) and *Semimembranosus* (SM). Cells from these two muscles were cultured and allowed to differentiate into myotubes. This process was studied using RNA-seq and morphological measurements across six time points. The main objective of this study was to describe the expression profile of genes during early muscle differentiation in Hanwoo and to find differentially expressed genes between LD and SM muscles that may be involved in modulating muscle fate. We found that the gene expression profile over time is similar in both muscles which indicates a highly conserved myogenic process. However, our results indicate that the two muscles differentiate at different rates and that 13 genes seem to be involved in determining the fate of the satellite cells into one muscle type or another. Identification of the biological triggers in the early stages of muscle development can be of value to understand the different characteristics of muscles in adult cattle.

## Results

### Morphological analysis

**Fig. 1:**
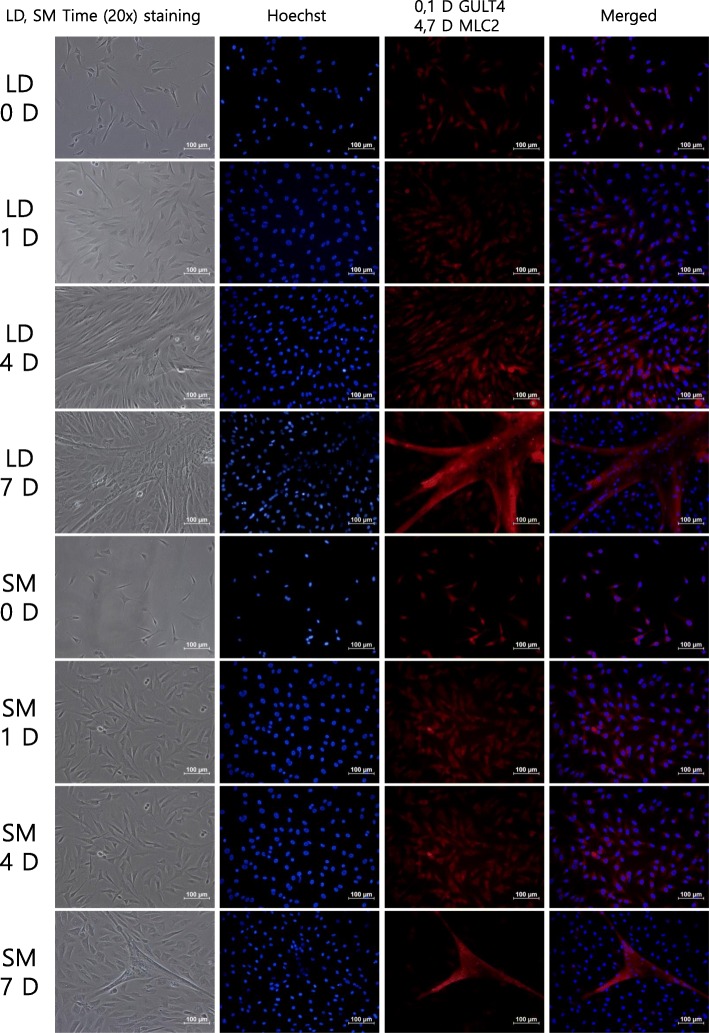
Phase contrast and immunohistochemistry of MSC during differentiation in bovine LD and SM tissues on days 0, 1, 4 and 7

**Fig. 2:**
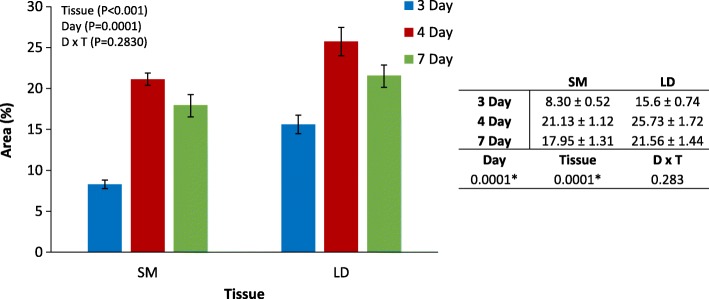
Differentiation indexes (area occupied by myotubes) on days 3, 4 and 7 in SM and LD

The in vitro bovine muscle satellite cells (MSC) proliferated until they reached 60–70% confluence after four or 5 days of culture. The MSC were then treated with the differentiation medium and this timepoint was taken as day 0 (Fig. 1). Differentiation of bovine myoblasts began between 2 and 4 days later. LD formed multinucleated myotubes with significantly higher differentiation indexes compared to SM at days 3, 4 and 7 (Fig. 2) which suggests a faster differentiation process in LD myoblasts. This faster differentiation in LD also hints at a faster proliferation rate in comparison to SM, however it was not measured in this study. On day 7, the myotubes of both muscles went through significant morphological changes by fusing to form mature multinucleated myotubes. There was also a significant reduction in the area occupied by the myotubes on day 7 in comparison to day 4 (Fig. 2). The differentiation indexes were calculated just for days 3, 4 and 7 since no myotubes were detected on day 2 (Fig. 2).

### Sequencing and alignment to the *B. taurus* genome

To characterize the gene expression profile during muscle differentiation, mRNA libraries were constructed at different stages of differentiation for LD and SM muscles. On average 80% of the paired reads mapped the *B. taurus* reference genome UMD3.1, from a mean value of 35,727,746 total reads per sample (Table 1 and additional details of the processed reads in Additional file 1). The principal components analysis of the gene expression showed that the differentiation stage was the primary source of variation and accounted for 81% of the variation; the differences in expression between the two muscle types explained a relatively small proportion of the variance (Additional file 2).

**Table 1:** Summary of RNA-seq reads

Muscle	Day	Total reads	Clean reads	Mapped reads
LD	0	64,653,898.67	32,326,949.33	82%
	1	84,792,260	42,396,130	80%
	2	83,802,156.67	41,901,078.33	80%
	4	77,402,347.33	33,542,207.33	80%
	7	59,158,033.33	29,579,016.67	80%
	14	59,413,041.33	29,706,520.67	80%
SM	0	68,553,754.67	34,276,877.33	83%
	1	80,691,319.33	40,657,234	80%
	2	83,065,964.67	41,532,982.33	81%
	4	79,723,010	39,861,505	82%
	7	62,983,208.67	31,491,604.33	81%
	14	62,921,703.33	31,460,851.67	81%

The values presented are the average of the three biological replicates for each muscle and time point

### Gene expression analysis

**Fig. 3:**
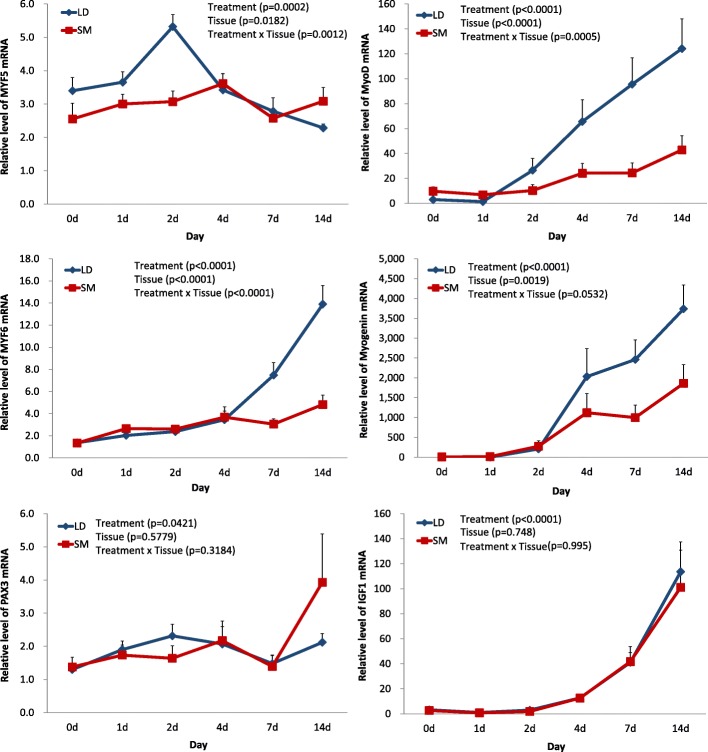
Expression of Myogenic Regulatory Factors and transcription factors in LD (blue lines) and SM (red lines) detected by qPCR. Error bars show standard errors of the mean of three biological replicates for three technical replicates

The stages of MSC differentiation observed in the histology aligned well with the qPCR time course expression of the Myogenic Regulatory Factors (MRF), *PAX3* (paired box 3) and *IGF1* (Insulin-like growth factor 1) in both muscles (Fig. 3). In LD, the expression of *MYF5 –* Myogenic Factor 5 (Fig. 3) rapidly increased on day 2, with a subsequent reduction in mRNA abundance from day 4 until day 14. This would be expected during the differentiation stage in which the myotubes were more abundant (Fig. 1). The expression in SM was relatively constant.

The expression of *MYOD* (myogenic differentiation 1) increased steeply after day 1 in LD. Expression levels were lower in SM and increases in expression were more gradual (Fig. 3). The increase of MYF6 (Myogenic Factor 6) mRNA is an indication of late differentiation which was observed from day 4 until day 14; here also a higher level of expression was observed in LD. *MYOG* (Myogenin) has a role in late differentiation [9] with the qPCR showing an increase in expression in both muscles from day 2 to day 14, and again a higher expression in LD. The higher expression of *MYOD*, *MYF6* and *MYOG* in LD during the stage of late differentiation coincides with the faster differentiation observed in this muscle compared to SM (Fig. 2). The expression of *PAX3*, which is high during proliferation of satellite cells, was consistently low during the differentiation stages in LD, however, there was an increase in its expression on day 14 in SM. The myogenic marker *IGF1* showed an increased expression from day 4 until day 14 (Fig. 3).

**Fig. 4:**
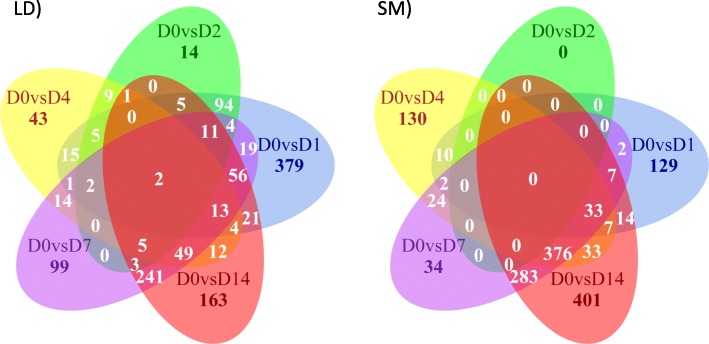
Venn diagram with the number of common DE genes (FDR < 0.05, abs(log_2_FC) ≥2) between contrast of each timepoint vs day 0 for LD and SM muscles

The number of differentially expressed genes across time was unsurprisingly large (Fig. 4). In relation to the baseline day 0, there were 631, 155, 175, 519 and 586 DE genes in LD, while in SM we found 204, 0, 615, 761 and 1154 DE genes at days 1, 2, 4, 7 and 14 respectively (the complete list of DE genes is provided in Additional file 3).

**Fig. 5:**
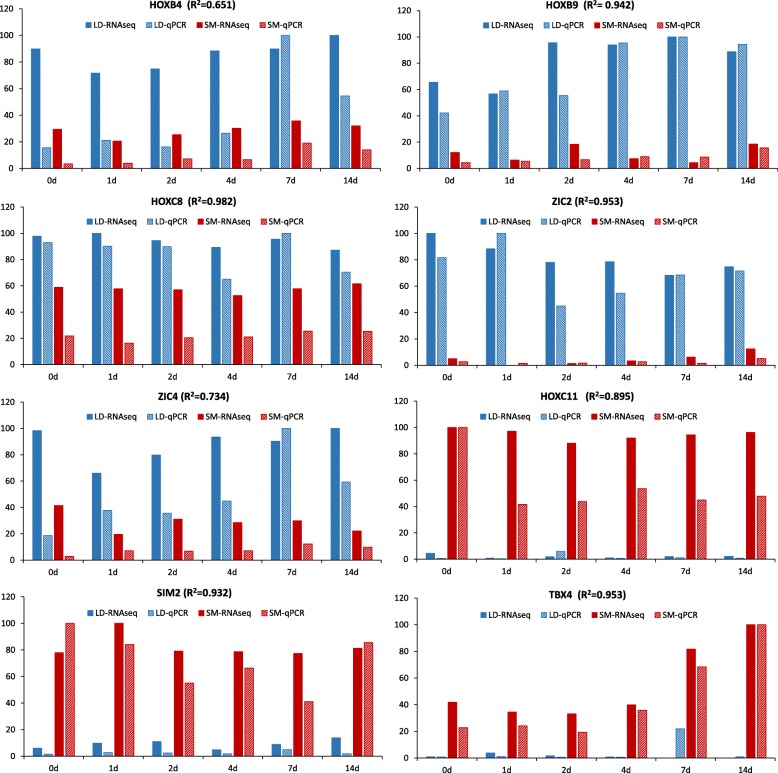
Expression profile of DE genes comparing RNA-seq (solid bars) and qPCR (checkered bars) results during differentiation of satellite cells from LD (blue bars) and SM (red bars) muscles. The R^2^ Pearson correlation is shown for each gene

Across all time points there were 13 genes differentially expressed between LD and SM (Additional file 3). The expression of the homeobox genes B2 (*HOXB2*), B4 (*HOXB*), B9 (*HOXB9*) and C8 (*HOXC8*), alongside forkhead box D1 (*FOXD1*), immunoglobulin-like and fibronectin type III domain containing 1 (*IGFN*), zinc finger members 2 (*ZIC2*) and 4 (*ZIC4*) were more abundant in LD, while the homeobox genes A11 (*HOXA11*) and C11 (*HOXC11*) plus paired-like homeodomain transcription factor 1 (*PITX1*), single-minded family bHLH transcription factor 2 (*SIM2*) and T-box 4 (*TBX4*) were more expressed in SM (Fig. 5 and Additional file 4). The consistent pattern of expression of these genes suggest that they could be involved in muscle fate differentiation into trunk or limb in cattle. Eight of these DE genes were randomly chosen for qPCR validation (Table 2). Significant differences in the relative expression levels were observed between muscles (*p* < 0.0001) confirming the results from the RNA-seq analysis.

**Table 2:** List of qPCR primers used to validate the expression results from the RNA-seq analysis

Gene	Sequence (5′-3′)
RPS9 (accession no. DT860044)	*Forward* - GAGCTGGGTTTGTCGCAAAA
	*Reverse* - GGTCGAGGCGGGACTTCT
	*Taqman probe* -6FAM-ATGTGACCCCGCGGAGACCCTTC-TAMRA
Myogenin (accession no. AF091714)	*Forward* - AGAAGGTGAATGAAGCCTTCGA
	*Reverse* - GCAGGCGCTCTATGTACTGGAT
	*Taqman probe* - 6FAM-CCCAACCAGAGGCTGCCCAAAGT-TAMRA
Myo D (accession no. AB110599)	*Forward* - AGGCCTTCGAGACGCTCAA
	*Reverse* - TGGCGTTGCGCAGGAT
	*Taqman probe* - 6FAM-CGCTGCACGTCTAGCAACCCAAACC-TAMRA
Myf 5 (accession no. NM_174116)	*Forward* - GGCTTTCGACACGCTCAAG
	*Reverse* - CATTCCTGAGGATCTCCACCTT
	*Taqman probe* - 6FAM-TGCACCACGACCAACCCTAACCAGA-TAMRA
MYF6 (accession no. NM_181811)	*Forward* - GGAGGTGGTGGAGAAGTAACTCA
	*Reverse* - GCAGGGAGGGTGGGATCTT
	*Taqman probe* - 6FAM-TCCGGACGTTCTCCACGGAGCA-TAMRA
IGF1 (accession no. NM_001077828)	*Forward* - GGTGAAGATGCCCATCACATC
	*Reverse* - GCTGGTGAAGGCGAGCAA
	*Taqman probe* - 6FAM-TCCTCGCATCTCTTCTATCTGGCCCTG-TAMRA
PAX3 (accession no. NM_001206818)	*Forward* - GGACAGCAGCTCTGCCTACTG
	*Reverse* - GAGGCACAAAGCTGTCTGTATAGC
	*Taqman probe* - 6FAM-CTCCCCAGCACCAGGCATGGA-TAMRA
HOXC11 (accession no. NM_001192873)	*Forward* - GCACTTACTACGTGCCTGAGTTCTC
	*Reverse* - TTGGGCCGGGTAGGGATA
	*Taqman probe* - 6FAM-CCCCAGGCCCCCTCTCGTCAG-TAMRA
SIM2 (accession no. XM_015462213)	*Forward* - CGAAGCTGAGAGCAAACCCTTA
	*Reverse* - CGCACTCCAGTTTGTCCATTT
	*Taqman probe* - 6FAM-CCCGCACAGCAGTACGGCTCG-TAMRA
HOXC8 (accession no. XM_002687232)	*Forward* - TCGCACCACGTCCAAGACT
	*Reverse* - TCTGCTGGTAGCCCGAGTTG
	*Taqman probe* - 6FAM-CTTCCACCACGGCACCTCGGG-TAMRA
HOXB9 (accession no. NM_001191186)	*Forward* - GGCAACCCCAGTTCCTCACT
	*Reverse* - TCCCCGGGTGACTTTGG
	*Taqman probe* - 6FAM-CCAACCTGCCTGTTCCTTCCCACA-TAMRA
ZIC2 (accession no. NM_001206366)	*Forward* - CAAGCAAGAGCTCATCTGCAAGT
	*Reverse* - TGAAAGTTTTGTTGCAGCTCTTCT
	*Taqman probe* - 6FAM-ATCGACCCCGAGCAGCTGAGTAACC-TAMRA
ZIC4 (accession no. XM_586391)	*Forward* - GCGCCTTTGCTCCAAAACT
	*Reverse* - ACGTGCTCCACGGTGACAT
	*Taqman probe* - 6FAM-TCAGCACCATGCACGAGCTGGTC-TAMRA
TBX4 (accession no. NM_001192193)	*Forward* - GATGTTCCCCAGCTACAAGGTAA
	*Reverse* - CGATGTCGATCAGCAGGATGT
	*Taqman probe* - 6FAM-CACGGGCATGAACCCCAAGACC-TAMRA
HOXB4 (accession no. NM_001078114)	*Forward* - AGTGTTTTGGCCACGGTAACA
	*Reverse* - CGGCCCCAAGGTGGAA
	*Taqman probe* - 6FAM-CTTCCCCCTCCATGCCCGTTCA-TAMRA

### Functional analysis of differentially expressed genes

The DE genes resulting from the time course analysis were used for a Gene Ontology (GO) and pathway enrichment analysis. Enriched GO terms (Table 3) for *Cellular components* included *extracellular space*, *contractile fiber*, *myofibril*, *sarcomere*, *actin cytoskeleton* and c*ytoskeletal part*. For *Biological process*es, some of the enriched terms were *cell cycle*, *cell proliferation*, *cytoskeleton organization* and *muscle structure development*. *Calcium ion binding*, *receptor activity* and *cytoskeletal protein binding* were enriched terms in the *Molecular functions* domain. The full list of enriched ontology terms is presented in the supplementary material (Additional file 5).

**Table 3:** Gene Ontology of the differentially expressed genes in LD and SM muscles for days 1, 7 and 14 in relation to day 0. MF: molecular function; CC: cellular components; BP: biological process. Number of DE genes that are up-regulated (↑) and down-regulated (↓)

		Day 1		Day 7		Day 14	
		*Term*	*DE*	*Term*	*DE*	*Term*	*DE*
*LD*	MF	Calcium ion binding	↓19	Cytoskeletal protein binding	↑7↓8	Receptor activity	↑15↓2
		Transmembrane receptor activity	↓15	Double-stranded DNA binding	↑4↓6	Cytoskeletal protein binding	↑9↓8
		Hormone activity	↑1↓7	Microtubule binding	↓7	Protein kinase binding	↑6↓5
						Peptidase inhibitor activity	↑4↓2
	CC	Extracellular space	↑ 4↓31	Cytoskeletal part	↑5↓20	Cytoskeletal part	↑7↓16
		Myofibril	↓25	Myofibril	↑12	Myofibril	↑13
		Sarcomere	↓22	Microtubule	↑12	Sarcomere	↑12
		Actin cytoskeleton	↓17	Sarcomere	↑11	Microtubule organizing center	↑1↓11
		I band	↓13	I band	↑7	I band	↑6
	BP	Regulation of multicellular organismal process	↑1↓37	Cell cycle	↑1↓33	Cell cycle	↑3↓29
		Immune system process	↑1↓27	Cytoskeleton organization	↑5↓15	Phosphorylation	↑13↓15
		Muscle structure development	↓17	Protein phosphorylation	↑6↓13	Cell proliferation	↑9↓16
		Actin filament-based process	↓14	Cell proliferation	↑5↓13	Muscle structure development	↑13↓3
		Muscle cell differentiation	↓11	Microtubule-based process	↓15	Negative regulation of proteolysis	↑6↓4
*SM*	MF			Cytoskeletal protein binding	↑12↓10	Receptor binding	↑15↓16
				Peptidase inhibitor activity	↑5↓4	Receptor activity	↑27↓3
				Protease binding	↑4↓2	Cytoskeletal protein binding	↑17↓12
						Peptidase inhibitor activity	↑7↓3
	CC	Contractile fiber	↓10	Cytoskeletal part	↑23↓13	Extracellular space	↑34↓13
		Sarcomere	↓9	Extracellular space	↑24↓9	Cytoskeletal part	↑18↓24
		Myofibril	↓9	Myofibril	↑20	Myofibril	↑23↓1
		Actin cytoskeleton	↓9	Sarcomere	↑18	Sarcomere	↑21↓1
		I band	↓5	Microtubule	↑1↓12	I band	↑12
	BP	Muscle structure development	↓7	Cell cycle	↑5↓34	Immune system process	↑36↓10
		Regulation of muscle system process	↓5	Cytoskeleton organization	↑10↓16	Cell cycle	↑6↓36
		Striated muscle tissue development	↓5	Inflammatory response	↑12↓3	Cell proliferation	↑15↓23
		Regulation of muscle contraction	↓4	Muscle organ development	↑12	Cytoskeleton organization	↑16↓18
				G2/M transition of mitotic cell cycle	↑1↓4	Muscle structure development	↑21↓5

**Fig. 6:**
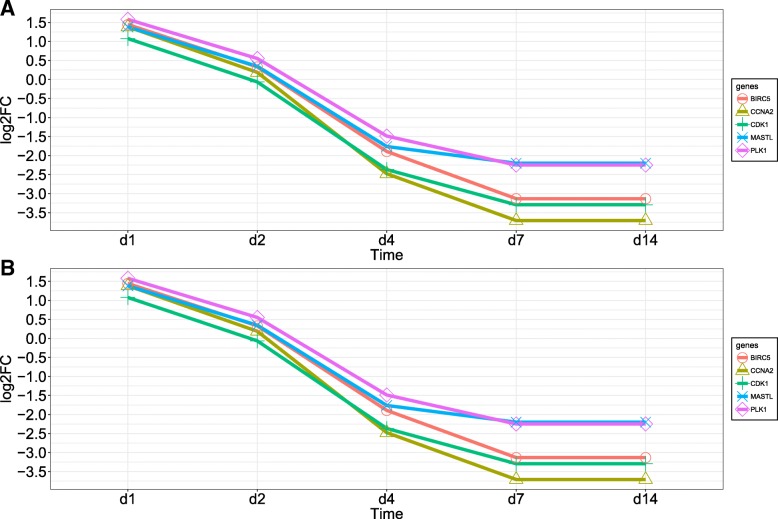
Differentially expressed genes involved in the GO term *G2/M transition of mitotic cell cycle* for **a**) LD and **b**) SM

At the beginning of the differentiation (day 1) most genes involved in any enriched GO term were down regulated in both muscles, which is consistent with the morphological changes observed in the histology. However, due to the structural changes observed when myoblasts differentiated into myotubes (days 7 and 14 in Fig. 1), more genes, especially those involved in cellular component terms like microtubule and sarcomere were up regulated (days 7 and 14, Table 3). The down regulation of genes involved in *cell cycle*, *G2/M transition of mitotic cell cycle* and *cell proliferation* in differentially expressed genes from days 7 and 14 corresponds to the shift from proliferation to differentiation that was observed in the cultures (Fig. 1). The proportion of DE genes changed mainly due to the advancement of differentiation. To get a better understanding of the shift from proliferation to differentiation, we plotted the log_2_-fold change of the genes involved in the term *G2/M transition of mitotic cell cycle* (Fig. 6). These genes were slightly upregulated at day 1 with a subsequent drop in abundance until day 4 and then low expression levels were maintained until day 14 in both muscles.

The main enriched pathways related with muscle differentiation were: *complement and coagulation cascades*, *cardiac muscle contraction*, *calcium signaling pathway*, *cell cycle* and *DNA replication*. The enriched pathways from the differentially expressed genes are reported for each muscle and time point in the supplementary material (Additional file 6).

## Discussion

Multiple genes have been identified in model organisms as being important to control the myogenic process. However, this is the first in vitro study to apply a time-series RNA-seq analysis to characterize the transcriptomic differences that occur during the differentiation processes of two distinct bovine MSC depots. Previous studies on bovine MSC have focused on the transcriptional differences in muscle proliferation rates [8], the last stage of differentiation or trans-differentiation of satellite cells [13] and the comparison of muscles [14]. Moreover, most studies have focused on the transcriptional differences between muscles [15], fat [16] or breeds [17] of adult animals.

### Transcriptional differences between LD and SM differentiation

The myogenic process during embryonic development can be approximated by in vitro differentiation of MSC to provide us with a better understanding of the developmental differences between different muscles. We identified consistent differences in the expression of 13 genes (Fig. 5) by comparing *Longissimus dorsi* (LD) and *Semimembranosus* (SM) muscle satellite cells during the differentiation process which suggests that they could be involved in driving muscle compartmentalization. Up regulation of *HOXB2*, *HOXB4*, *HOXB9*, *HOXC8*, *FOXD1*, *IGFN1*, *ZIC2* and *ZIC4* genes in LD suggest their role in lineage development of MSC into trunk muscles. On the other hand, the higher expression of the genes *HOXA11*, *HOXC11*, *PITX1*, *SIM2*, and *TBX4* in SM could be involved in the development of MSC into limb muscles.

We did not test this in the study, but it is possible that epigenetic regulation during the embryonic development of LD and SM muscles as well as neighboring signals could have imprinted the MSC with specific methylation patterns that resulted in the observed differences in expression patterns during in vitro differentiation. Another study found similarly divergent expression in human abdominal adipose cells where the genes that showed higher expression were generally hypomethylated in the CpG regions and genes with lower expression had CpG regions more associated with hypermethylation when compared to gluteal cells [18]. In the same study, the gene *HOXC11* was more expressed in the gluteal cells which lends support to its potential role in the development of limb structures. Epigenetic imprinting is essential for muscle regeneration [19] and to guide the asymmetric division of SC into specific cellular fates [20]. However, methylation studies with LD and SM MSC will be required to understand if there truly are epigenetic differences between these bovine muscle types and what role they play in muscle differentiation.

The expression of HOX seems to be responsible for the correct development of muscles and regulation of muscle-specific genes in mature muscle tissue [21]. In chicken embryos the *HOXA11* protein was found in myogenic precursor cells in the early limb bud and its expression gradually reduced as development progressed [22]. Also in chicken embryos, this gene is expressed at somite level 33–34 and overlaps with the hindlimb bud [23]. Forced expression of *HOXA11* reduces the abundance of *MYOD* in the limb of embryo chickens and a similar repression of *MYOD* was observed in C2C12 cells transfected with *HOXA11* [24]. The inhibiting action of *HOXA11* upstream of *MYOD* could explain the higher expression of *MYOD* (Fig. 3) observed in LD in relation to SM due to the higher expression of *HOXA11* in SM. After day 1, in both muscles, the expression of *MYOD* increased after the expression of *HOXA11* went down (Additional file 4). These results suggest that *HOXA11* can control the speed of differentiation depending of the origin of the muscle (in this case it promoted a delay in the differentiation of SM) which will guide the determination of different muscle types.

Genes from the *HOXC* cluster (Homeobox C Cluster) were also reported in muscles where myoblast and myotubes showed differential methylation and that these genes present active promoters and enhancer domains that contain *MYOD* binding sites [25]. *HOXC8* and *HOXC11* were reported to be involved in the regulation of osteogenesis [26]. Recently it was suggested by [21] that only a subset of muscles may require *HOXC8* protein for full activation of muscle-specific gene expression. In chicken and mouse embryos, the expression of *HOXC8* lies in the trunk region posterior to the forelimb [23]. In mice embryos (E11.5) the gene *HOXC11* was expressed on the rostral side of somite 27, the region involved in the development of hind limb buds [27]. However, the in-situ hybridization in chick embryos showed the location of *HOXC11* at somite 36–37 mapping to the seventh sacral vertebrae at the posterior edge of the hindlimb [23].

A study in chicken found that *TBX4* seems to be involved in controlling limb identity [28] which supports our conclusion that *TBX4* is involved in the regulation of SC differentiation into SM muscle. However, in mice embryos the gene *TBX4* seems to regulate muscle and tendon patterning but has not been implicated in their development [29]. *SIM2* also upregulated in SM has been shown to be expressed in mice and chicken embryonic limbs and with a higher expression in the ventral limb myoblast [30].

In adult mice, the induced over expression of *PITX1* led to significant body weight loss and muscle mass reduction which was primarily caused by muscle atrophy [31]. Using retroviral constructs for *PITX1*, *PITX2* and *PITX3* in satellite cells from mice, Knopp et al. [32] observed that the increased expression of the paired like homeodomain genes (*PITX)* suppressed the proliferation of myoblasts and increased the fusion into multinucleated myotubes during differentiation. In mice, Marcil et al. [27] generated double mutants for *PITX1* and *PITX2* – both mutant embryos lost some hind limb features and had smaller hind limb buds compared to the wild type [27]. In the same study, the expression of *TBX4* (assessed by in situ hybridization) decreased around the hind limbs suggesting a role in the specification of hind limbs identity through a cascade that involved *TBX4* and *PITX1* as an upstream regulatory gene [27].

The deficiency of *HOXB4* reduces the capacity of hematopoietic stem cells to proliferate [33]. In myogenic progenitor cells the sub-region that contains *HOXB4*, *HOXB5*, *HOXB6*, *HOXB7* and *HOXB-AS3* is hypermethylated [25]. During chicken embryo development there are individual cells that express only a subset of the genes depending of the rhombomere of origin. The expression of *HOXB4* was detected in rhombomere 7 while rhombomeres 4 and 5 express *HOXB2* [34]. An in-situ hybridization study in chicken and mice associated the expression of *HOXB4* with the anterior cervical vertebrae while *HOXB9* was mapped to the posterior trunk [23].

Pan et al. [35] used mice to show that the knockdown of *ZIC2* resulted in a delay in the activation of *MYF5* with a subsequent delay in *MYOD*, but the expression of *PAX3* was not affected by the absence of *ZIC2*. This is comparable to our results, the expression of *ZIC2* was higher in LD which could explain the increase of *MYF5* in this muscle at days 0, 1 and 2 and subsequently the faster increase of *MYOD* in LD (Fig. 2). *ZIC4* also had a higher expression in LD. During mouse embryogenesis the expression of *ZIC4* is in the dorsal midline of the forebrain and in the dorsal spinal neural tube at E12.5, while at the mid-trunk level *ZIC4* is restricted to the most dorsal part of the sclerotome and the dorsomedial dermomyotome [36].

*FOXD1* is another gene that has been associated with myogenesis control by regulating the expression of *MRF*. During the embryogenesis of flounder, the expression of *FOXD1* at the 6 somite stage was found in adaxial cells and progenitor cells of the forebrain, midbrain and kidney [37]. Injection of mRNA *FOXD1* reduced the speed of embryo development and the expression of *MYOD* in the somites, but the adaxial cells were not affected [37]. The negative effect of *FOXD1* on *MYOD* was not clearly observed in our results (Fig. 3, Additional file 4), but there was however a negative correlation between the expression of these genes (− 0.39). The correlation is stronger in LD (− 0.65) than in SM (− 0.17). The higher expression of *FOXD1* in LD together with the possible dampening effect on the expression of *MYOD* suggests that *FOXD1* may also be involved in muscle type differentiation.

### Myogenic development of bovine muscle satellite cells

The bovine MSC presented a pattern of expression in the *MRF* genes similar to C2C12 mouse cells during differentiation. As in other studies, *MYOD*, *MYF6* and *MYOG* were up-regulated during the differentiation of myoblast into myotubes. This was expected since *MYOG* reportedly regulates the fusion of myoblast into multinucleated muscle cells and *MYF6* is expressed only in the last stage of the C2C12 mouse cell during differentiation [38]. A gene expression analysis of the goat muscle *rectus abdominis* reported that *MYOD*, *MYOG* and *MYF6* were up-regulated while *MYF5* was down-regulated in differentiated myotubes compared with proliferative myotubes [39]. It has also been reported that *MYF6* began to be expressed during differentiation [40] and that the protein levels of MYOD, MYF5 and MYOG increased at day 10 of differentiation [12].

We found the same pattern in the transcription levels of *MYOG*, *MYOD* and *MYF6* for both muscles (but with a significantly higher expression in LD than in SM) indicating that the formation of multinucleated myotubes requires an increase in the expression of *MYOD*, *MYOG* and *MYF6*. Respectively, mRNA abundance levels for each gene started to increase on days 1, 2 and 4 and then remained high in the differentiated myotubes on day 14 (Fig. 3). In the case of *MYF5*, we observed a higher expression in LD on days 0, 1 and 2 with a slight reduction after day 2 in LD and day 4 in SM. In C2C12 myoblasts it was observed that the down-regulation of *MYOG* levels increased the levels of *MYF5* [38] and in *MYF5* null mice the differentiation is delayed at an early stage of regeneration [40]. In cattle, the down regulation of *MYF5* seems to reduce the proliferation of myoblasts because it maintains satellite cell pools quiescent and reduces the number of activated satellite cells [8]. Together, the expression of these genes could explain the faster developmental differentiation of LD and its higher number of myotubes per area (Fig. 2).

The expression of *PAX3* was low but constant, suggesting that some myoblasts were still proliferating as previously reported in mice SC [41] or that it plays a role during the differentiation of bovine muscle. With *IGF1*, its mRNA abundance increased on days 4, 7 and 14, which was expected as the IGF1 hormone is one of the most important muscle growth factors secreted by myocytes [9].

### Functional analysis

Overall, the GO enrichment analysis showed that most of the over-represented terms and genes expressed during myoblast differentiation were similar regardless of muscle type. We observed more differences in the enriched GO terms during the end of proliferation (day 0) compared to myoblast differentiation (days 4 and 7). At the beginning of the experiment, the terms *cell cycle*, *proliferation* and *G2/M transition of mitotic* (Fig. 6) had genes with higher expression, which agrees with the proliferation events that were occurring in the myoblasts. However, the expression of genes involved in these terms started to decrease in the other days due to the shift from cell division to cell differentiation, which is similar to results from previous studies in bovine [5].

From day 2 and onwards the main enriched terms were *actin cytoskeleton*, *myofibril assembly* and *muscle cell differentiation* (Table 3). These changes agreed with the progress of myoblast differentiation and reflected the changes in cellular structure at the cytoskeleton level during myoblast fusion; similar GO terms were already previously reported [12]. Enriched cellular component terms *myofibril* and *contractile fiber* are in line with the work of Tripathi et al. [39]. The term *inflammatory response* was found significant on days 1 and 4 in LD and on days 4, 7 and 14 in SM, consistent with He and Liu [5] who connected it with a muscle regenerative process.

The enrichment of genes (actinin alpha 2 – *ACTN2*, calpain 3 – *CAPN3*, myosin light chain 1 – *MYL1*, myosin light chain 2 – *MYL2*, complement C1s – *C1S* and troponin C1 – *TNNC1*) involved in calcium ion binding increased during the differentiation of myoblast into myotubes. During this stage, two main events require an increase in the flow of calcium (Ca++): 1) the fusion of myoblasts and 2) the development and organization of the contractile apparatus. In previous studies, it was suggested that the concentration of calcium ions influenced the fusion of myoblasts and therefore the number of nuclei in the myotubes of chicken [42]. On one side, Ca++ uptake increases before the fusion of the cytoplasmic vesicles with the cell membrane and acts as an intracellular stimulus [43]. On the other side, muscle contraction requires an increase in calcium-dependent cyclin proteins in order to increase the influx of Ca++ [44].

## Conclusion

The use of satellite cells from newborn Hanwoo calves appears to be a good model to study embryonic myogenesis in muscle. Our findings provide evidence that the differential expression of *HOXB2*, *HOXB4*, *HOXB9*, *HOXC8*, *FOXD1*, *IGFN1*, *ZIC2*, *ZIC4*, *HOXA11*, *HOXC11*, *PITX1*, *SIM2* and *TBX4* genes could be involved in the differentiation of *Longissimus dorsi* and *Semimembranosus* muscles. These genes seem to modulate the muscle fate of the satellite cells during myogenesis through a differential expression profile that also controls the expression of some myogenic regulatory factors (*MYOD* and *MYF5*). It is possible that the different profiles observed in the cell cultures are due to the origin of the satellite cells that where epigenetically imprinted during the embryonic development of *Longissimus dorsi* and *Semimembranosus*. However, epigenetic studies with LD and SM MSC will be required to understand if there truly are epigenetic differences between these bovine muscle types and what role they play in muscle differentiation.

## Methods

### Isolation of bovine satellite cells

Satellite cells were isolated from *Longissimus dorsi* (LD) and *Semimembranosus* (SM) muscle samples of three unrelated Korean Hanwoo newborn calves to investigate gene expression changes during differentiation of myoblasts fusing into multinucleate myotubes. Tissues were collected from calves that died spontaneously during delivery from Hanwoo mothers belonging to the Hanwoo Research Institute’s cattle herd (NIAS, Korea). The study was approved under the animal care and use protocols 2015–112 and 2018–319 by the Institutional Animal Care and Use Committee of the National Institute of Animal Science, Korea.

Satellite cells were then excised from the LD and SM tissues as previously described in [45, 46]. Briefly, the muscle samples were cut into 600–700 g pieces and transferred into a 1000 ml beaker with phosphate buffered saline (PBS). The samples were then transferred to a sterile area in a laboratory and the connective tissue was removed in the tissue culture hood. The muscles were then chopped up with sterile scissors and placed in satellite cell isolation buffer for incubation. Samples (250 g) were incubated with 300 ml of 0.1% pronase in Earl’s Balanced Salt Solution (EBSS) for 1 h at 37 °C with frequent mixing. Following incubation, the mixture was centrifuged at 1500 x g for 4 min and the supernatant was discarded. The pellet was suspended in phosphate-buffer saline (PBS; 140 mM NaCL, 1 mM KH_2_PO_4_, *3 mM KCl, 8 mM Na*_*2*_*HPO*_*4*_) and the suspension was centrifuged at 500 x g for 10 min. The resulting supernatant was centrifuged at 1500 x g for 10 min to pellet the mononucleated cells. The PBS was washed, and the differential centrifugation process was repeated another two times. Afterwards, the resultant mononucleated cell preparation was suspended in cold (4 °C) Dulbecco’s Modified Eagle Medium (DMEM) that contains 10% fetal bovine serum (FBS) and 10% (vol/vol) dimethylsulfoxide (DMSO) and frozen for subsequent studies.

### Satellite cell culture

Hanwoo satellite cells from both LD and SM muscles were separately plated on 225-cm^2^ culture plates percolated with reduced growth factor basement membrane Matrigel, diluted 1:10 (vol/vol) with DMEM containing 10% FBS and incubated at 37 °C, 5% CO_2_ in a water-saturated environment. Plating density was 2 × 10^4^ cells/ml so that all cultures were approximately 60 to 70% confluent after the incubation period. This ensured that the cell proliferation rate was not affected by contact inhibition. Cultures were fed at 48 h with DMEM containing 10% FBS.

### Differentiation treatment and cell sample collection

Cultures that reached confluence (considered as day 0) were stimulated to differentiate with an induction medium that consisted of DMEM with 3% horse serum for 14 days. Plates were triplicated for each sample for the RNA-seq and morphological analyses. Samples from the LD and SM satellite cell cultures from each of the three biological replicates were scrapped from the dishes at six time points (days 0, 1, 2, 4, 7 and 14) and transferred to NuncTM Cryobank Vial System tubes with 1 ml capacity (Thermo Fisher Scientific, Carlsbad, CA, USA). Samples were carefully frozen with liquid nitrogen and then stored at − 80 °C until RNA extraction.

### Histological procedures

We performed Hematoxylin staining to determine the stages of differentiation and the differentiation index (at days 3, 4 and 7) of the culture plates for each muscle. The differentiation index is defined as the area, in percentage, occupied by the myotubes in the cultures. This index was calculated by calculating the area occupied by myotubes in at least five photographs taken from random fields of each plate. Photographs were taken using a Nikon NIS-elements (version 4.0; F-package) and Nikon Mi2 microscope with 20X phase-contrast objective. Differences in the index between LD and SM muscles at each time point were tested with an analysis of variance for a model fitting time (day), tissue and the interaction between time and tissue. *P*-values < 0.05 were considered significantly different.

### Immunohistochemical procedures

The differentiation of LD and SM muscle satellite cells (MSC) was evaluated by immunohistochemistry using a glucose transporter 4 (GLUT4; Abcam) antibody at days 0, 1 and 4 and a Myosin Light Chain (MLC; Abcam) antibody at day 7. Cultured cells were permeabilized using Triton 100 with PBS. Sections were blocked with a 10% goat serum in PBS, after which they were incubated with the GLUT4 and MLC antibody (20 μl/mL, Abcam) for 1 h. After washing with PBS for 5 min, cultures were treated with the second antibody for another hour at room temperature and then, after PBS washing, stained with Hoechst (1.5μg/ml) for 1 h. A negative control experiment was also carried out by omitting the antibody. Photographs were taken using a Nikon NIS-elements (version 4.0; F-package) and Nikon Mi2 microscope with 20X phase-contrast objective and a red filter. Nikon TRITC was used for wavelength detection from excitation 540 to barrier 605. The wavelength of secondary antibody (Alexa 568; Abcam 175,696) was between 578 nm excitation and 603 nm emission.

### RNA extraction and sequencing

RNA from the satellite cell cultures of the three Hanwoo calves was extracted from the LD and SM muscles for the six time points (days 0, 1, 2, 4, 7 and 14). Total RNA was isolated with TRIzol following the manufacturer’s protocol (Invitrogen, Carlsbad, CA, USA). RNA quality and quantity were assessed using an automated capillary gel electrophoresis on a Bioanalyzer 2100 with RNA 6000 Nano Labchips according to the manufacturer’s instructions (Agilent Technologies Ireland, Dublin, Ireland). High-quality RNA (RNA integrity number > 7.2) was processed using the Illumina TruSeq RNA Sample Preparation Kit (Illumina, San Diego, CA, USA) following the manufacturer’s procedures. The final individual RNA-Seq libraries were constructed using the Illumina Hiseq2000 platform, which created 100 bp/paired-end (PE) sequence reads.

### RNA-seq analysis

Quality of the RNA sequence data was evaluated with *FastQC* v0.11.3 [47]. Low quality bases and adaptor sequences were removed with *Trimmomatic* v0.33 [48]. Reads were mapped to the reference genome (*Bos taurus* Ensembl UMD3.1) using *Bowtie2* v2.2.6 [49]. Reads that mapped to multiple sites, single reads and unmapped reads were excluded from the analysis. Downstream analyses were performed with the statistical programming language R [50] and various R packages. *GenomicFeatures* v1.22.13 [51] and *GenomicAlignments* v1.6.3 [51] were used to annotate and count the reads. Principal Components Analysis (PCA) was used to evaluate the clustering of the samples (time points and muscle types). *edgeR* 3.12.0 [52] was used for the analysis of differentially expressed (DE) genes by fitting a generalized linear model with a negative binomial distribution to model the data effects for time and tissue. RNA composition was normalized with the scaling factors from the trimmed means of the M-values using *edgeR*. Gene expression differences between muscles (LD vs SM) at each timepoint were evaluated as well as the changes in expression over time for each muscle in relation to day 0. Genes were considered as differentially expressed for a log fold change ≥2 (or ≤ 2, depending on the order of the contrast) [52] and a false discovery rate (FDR) adjusted *p*-value < 0.05. The differentially expressed genes found in the study were tested with *ClusterProfiler* v2.5.5 [53] for Gene Ontology (GO) and Pathway enrichment analyses. This R package uses data from the Gene Ontology and the Kyoto Encyclopedia of Genes and Genome (KEGG) databases to test for over-representation of DE genes in ontologies and pathways. For both analyses we considered terms and pathways as significant for a cutoff q-value < 0.05 to reduce false discovery rates due to multiple testing. The DE genes in the overrepresented terms had their expression across time (fold change ratio relative to day 0) graphed to evaluate the dynamics of expression changes from day 0 to 14. We also used *VennDiagram* v1.6.18 [54] to visualize overlap of differentially expressed genes across time points for each muscle. The background set of genes for both enrichment analyses consisted of the bovine genes expressed in this study after quality control.

### qPCR validation of differentially expressed genes

From the list of differentially expressed genes, eight were randomly selected for validation using quantitative PCR (qPCR). Expression of the MRF genes was also measured with qPCR due to their known role in myogenic processes. Total RNA was isolated from LD and SM muscle satellite cell cultures at six time points (days 0, 1, 2, 4, 7 and 14) from three independent biological replicates. Reverse transcription to cDNA was performed using the commercial kit SuperScript III First-Strand Synthesis Supermix (Thermo Fisher Scientific, Carlsbad, CA, USA) following the manufacturer’s instructions. The primer sequences are listed in Table 2.

The measurement of the relative quantity of the cDNA of interest was performed using TAMRA PCR Master Mix (Applied Biosystems) with the appropriate forward and reverse primers and 1 μL of the cDNA mixture. We used the Sequence Detection System (Applied Biosystems) with the thermal cycling parameters recommended by the manufacturer (40 cycles of 15 s at 95 °C and 1 min at 60 °C). Relative expression was quantified with the 2^-ΔΔCt^ method [55]. All sample values were normalized against the *ribosomal protein S9* (*RPS9*) gene and expressed in arbitrary units. Titration of MRF mRNA primers against increasing amounts of cDNA gave linear responses with slopes between − 2.8 and − 3.0. To reduce the effect of assay-to-assay variation in the PCR assay, all values were calculated relative to a calibration standard run on every real-time PCR assay.

We used an ANOVA analysis to test the effects of tissue, time and the tissue by time interaction. Data were analyzed as a 2 (tissue) × 6 (time) factorial arrangement of treatments in a randomized complete block design with the PROC MIXED procedure (SAS Inst. Inc., Cary, NC). Main effects and interaction means were separated (*P* < 0.05) with the LSMEANS procedure of SAS. *P*-values came from the ANOVA analysis of the linear model:

$$ \mathrm{y}\sim \upmu +\mathrm{treatment}+\mathrm{tissue}+\mathrm{treatment}\times \mathrm{tissue}+\mathrm{e} $$

where treatment indicates time series for days 0, 1, 2, 4, 7 and 14 (6 levels) and tissue is LD and SM (2 levels).

## Additional files

Additional file 1: **Table S1.** Summary of preprocessing of RNA-seq reads and mapping steps for each sample. (DOCX 17 kb)

Additional file 2: **Figure S1.** Principal component analysis of the expression profiles for each time point and muscle type. (PDF 60 kb)

Additional file 3: **Table S2.** List of differentially expressed genes from the contrast LD vs SM and contrasts at each time point. (XLSX 380 kb)

Additional file 4: **Figure S2.** Expression profile of LD and SM in log_2_ (cpm). (PDF 165 kb)

Additional file 5: **Table S3.** Gene Ontology results for each of the time contrasts. (XLSX 73 kb)

Additional file 6: **Table S4.** Pathway enrichment analysis of contrasts over time for LD and SM. (XLSX 26 kb)

## Abbreviations

BP: Biological process
CC: Cellular components
CPM: Copies per Million
DE: Differentially expressed
LD: *Longissimus dorsi*
MF: Molecular function
MRF: Myogenic regulatory factors
MSC: Muscle satellite cells
SM: *Semimembranosus*
