# Supplementary material for: Gene expression of Hanwoo satellite cell differentiation in longissimus dorsi and semimembranosus
Source: BMC Genomics. 2019 Feb 26;20:156. doi: 10.1186/s12864-019-5530-7 (PMC6390542; doi:10.1186/s12864-019-5530-7)
Supplement: Supplementary file 1 — Table S1. Summary of preprocessing of RNA-seq reads and mapping steps for each sample. (DOCX 17 kb) [file 12864_2019_5530_MOESM1_ESM.docx]

**Supplementary Table**

**Table S1.** Summary of RNA-seq data

| **Muscle** | **Animal** | **Day** | **Total reads** | **Cleaned reads** | **Mapped reads** |
| --- | --- | --- | --- | --- | --- |
| LD | 1 | 0 | 69806844 | 34903422 | 82.34% |
| LD | 2 | 0 | 59846170 | 29923085 | 81.08% |
| LD | 3 | 0 | 64308682 | 32154341 | 82.27% |
| LD | 1 | 1 | 78649942 | 39324971 | 79.97% |
| LD | 2 | 1 | 91066954 | 45533477 | 79.48% |
| LD | 3 | 1 | 84659884 | 42329942 | 80.20% |
| LD | 1 | 2 | 75634576 | 37817288 | 80.58% |
| LD | 2 | 2 | 91189606 | 45594803 | 79.19% |
| LD | 3 | 2 | 84582288 | 42291144 | 79.76% |
| LD | 1 | 4 | 81190918 | 40595459 | 80.61% |
| LD | 2 | 4 | 79843476 | 39921738 | 77.82% |
| LD | 3 | 4 | 71172648 | 20109425 | 83.04% |
| LD | 1 | 7 | 69541392 | 34770696 | 79.92% |
| LD | 2 | 7 | 55351100 | 27675550 | 79.40% |
| LD | 3 | 7 | 52581608 | 26290804 | 80.32% |
| LD | 1 | 14 | 67459076 | 33729538 | 80.84% |
| LD | 2 | 14 | 56685992 | 28342996 | 79.58% |
| LD | 3 | 14 | 54094056 | 27047028 | 80.88% |
| SM | 1 | 0 | 65522002 | 32761001 | 81.01% |
| SM | 2 | 0 | 73700856 | 36850428 | 82.25% |
| SM | 3 | 0 | 66438406 | 33219203 | 84.63% |
| SM | 1 | 1 | 76197334 | 38098667 | 79.77% |
| SM | 2 | 1 | 83086186 | 41543093 | 79.94% |
| SM | 3 | 1 | 82790438 | 42329942 | 80.20% |
| SM | 1 | 2 | 82068166 | 41034083 | 83.32% |
| SM | 2 | 2 | 85892946 | 42946473 | 80.40% |
| SM | 3 | 2 | 81236782 | 40618391 | 80.51% |
| SM | 1 | 4 | 77252852 | 38626426 | 81.28% |
| SM | 2 | 4 | 84207802 | 42103901 | 84.96% |
| SM | 3 | 4 | 77708376 | 38854188 | 80.98% |
| SM | 1 | 7 | 57992502 | 28996251 | 83.55% |
| SM | 2 | 7 | 69056382 | 34528191 | 80.31% |
| SM | 3 | 7 | 61900742 | 30950371 | 80.52% |
| SM | 1 | 14 | 64042232 | 32021116 | 80.46% |
| SM | 2 | 14 | 68678636 | 34339318 | 80.55% |
| SM | 3 | 14 | 56044242 | 28022121 | 80.65% |
