# Supplementary material for: Gene expression of Hanwoo satellite cell differentiation in longissimus dorsi and semimembranosus
Source: BMC Genomics. 2019 Feb 26;20:156. doi: 10.1186/s12864-019-5530-7 (PMC6390542; doi:10.1186/s12864-019-5530-7)
Supplement: Supplementary file 2 — Figure S1. Principal component analysis of the expression profiles for each time point and muscle type. (PDF 60 kb) [file 12864_2019_5530_MOESM2_ESM.pdf]

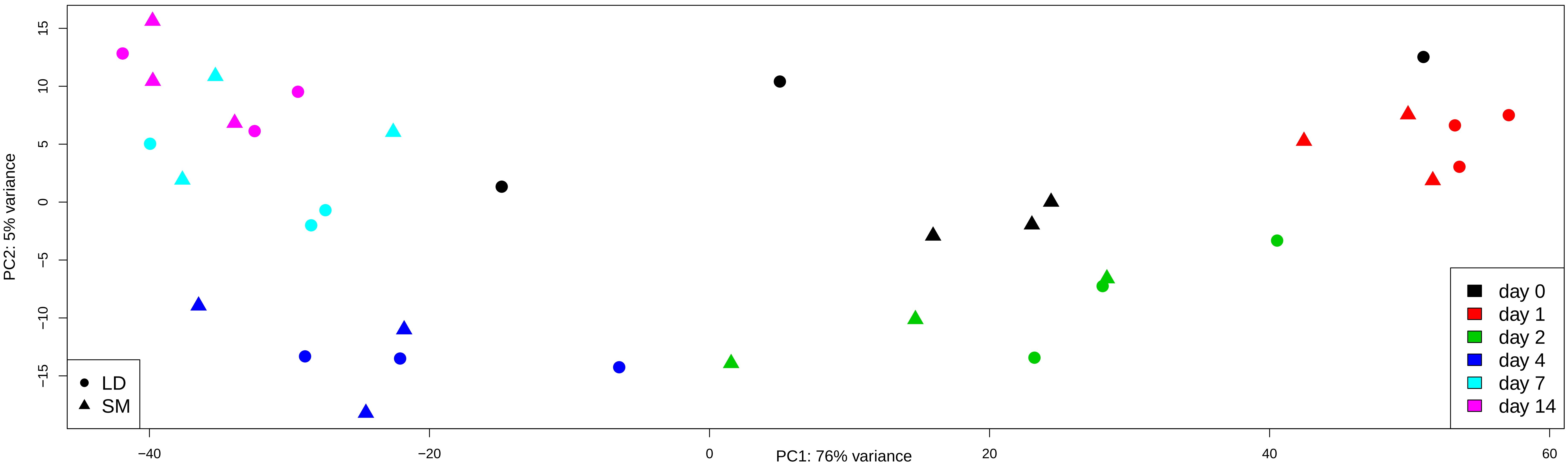

Figure S1. Principal component analysis of the expression profiles for each time point and muscle type
