# Supplementary material for: Gene expression of Hanwoo satellite cell differentiation in longissimus dorsi and semimembranosus
Source: BMC Genomics. 2019 Feb 26;20:156. doi: 10.1186/s12864-019-5530-7 (PMC6390542; doi:10.1186/s12864-019-5530-7)
Supplement: Supplementary file 4 — Figure S2. Expression profile of LD and SM in log2 (cpm). (PDF 165 kb) [file 12864_2019_5530_MOESM4_ESM.pdf]

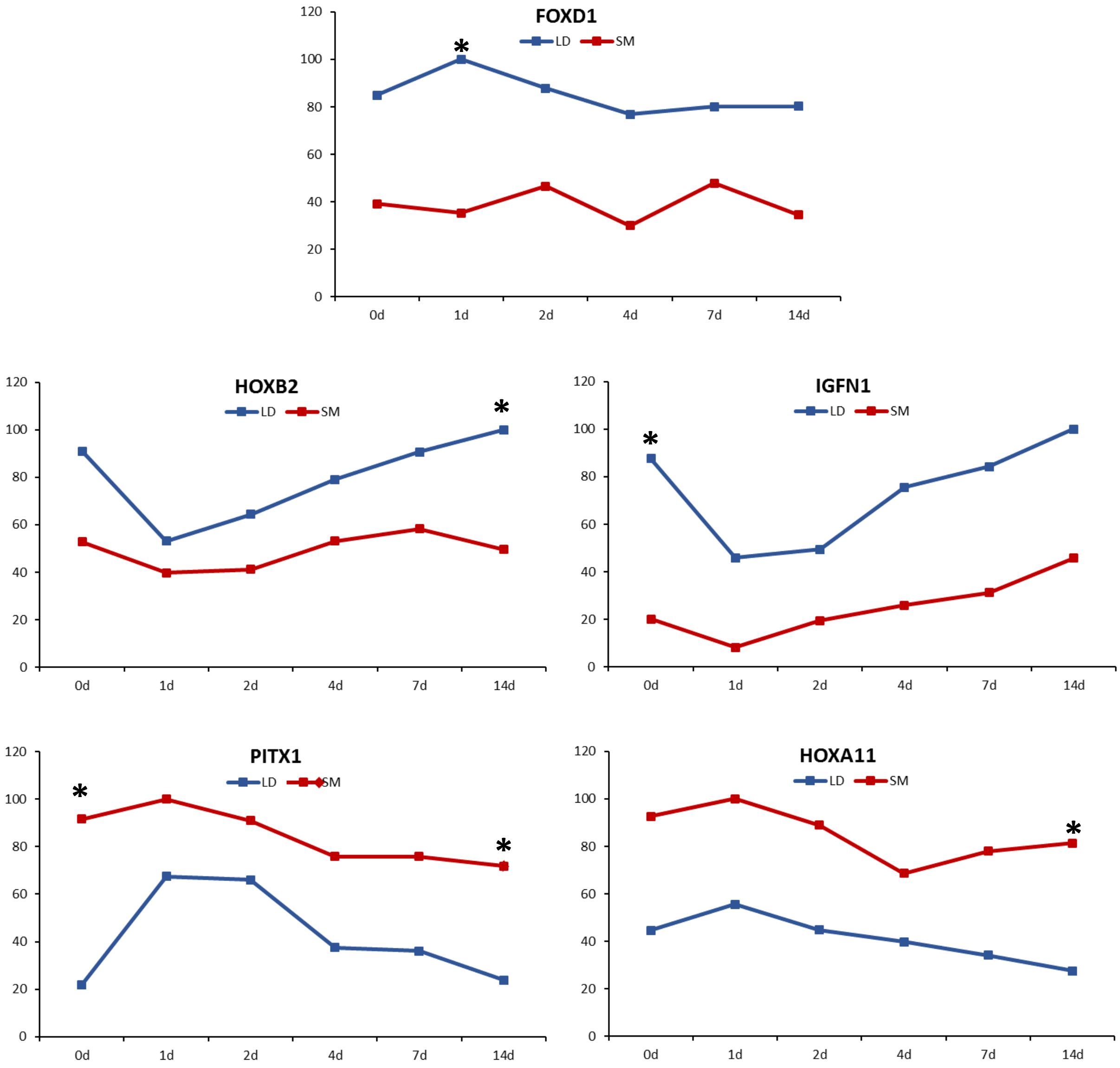

Figure S2. Expression profile of DE genes from RNA-seq results during differentiation of satellite cells from LD (blue lines) and SM (red lines). Plots show the ratio of the $\log_2$ of cpm for each muscle and time point.
